# Correction: Inequality of the crowding-out effect of tobacco expenditure in Colombia

**DOI:** 10.1371/journal.pone.0315977

**Published:** 2024-12-12

**Authors:** Juan Miguel Gallego, Guillermo Paraje, Paul Rodríguez-Lesmes

The affiliations for the second and third authors are switched. Guillermo Paraje is not affiliated with #1 but with #2: Escuela de Negocios,Universidad Adolfo Ibáñez, Diagonal las Torres, Peñalolén, Santiago, Chile. Paul Rodríguez-Lesmes is not affiliated with #2 but with #1: School of Economics, Universidad del Rosario, Calle, Bogotá, Colombia.
