# Supplementary material for: Inequality of the crowding-out effect of tobacco expenditure in Colombia
Source: PLoS One. 2024 May 21;19(5):e0303328. doi: 10.1371/journal.pone.0303328 (PMC11108158; doi:10.1371/journal.pone.0303328)
Supplement: S1 File — Appendix A. DataAppendix B. Robustness. (PDF) [file pone.0303328.s001.pdf]

Inequality of the crowding-out effect of tobacco expenditures in Colombia

Supplemental Material

Appendix A. Data

Quality of Life Survey

Our analysis uses household data from ECV 1997, 2003, 2008 and 2011. While these surveys do not include individual cigarette consumption, they measure total household expenditure on tobacco products during the previous seven days. Because this is a cross-sectional survey, we constructed a pseudo-panel using matching techniques for the purposes of this study.

Although expenditure data are used instead of consumption data, our estimates of prevalence obtained from ECV are similar to those based on individual-level data collected by specialized tobacco surveys. The prevalence of tobacco consumption in 2008 was around 17% for individuals aged 12 to 65 years of age. A household prevalence of 16.5% is estimated for the same year using the ECV 2008. The latter figure is expected to be lower as a result of the effect of aggregating several smokers into a single household unit

Sample Selection

- As data are collected from households, we used the sociodemographic characteristics of the household heads, such as education, gender, and age, to construct a pseudo-panel to assess smoking inequalities.

Imputation of expenditure data

- For missing values, both at the individual and household levels, we used mean values based on a household socio-economic classification at the national level, which is used for assigning taxes and subsidies for public utilities (*Estrato*)

Definitions of variables

- *Tobacco prices:* Although other tobacco products are available, tobacco expenditure in Colombia is mainly on cigarettes. A price per cigarette series was constructed using the annual cigarette-specific CPI index, and a reference price of COP 121 per cigarette in 2011 was derived from the average cigarette pack prices published by the Colombian Department of Statistics (DANE).

- *Consumption intensity:* We created a smoking-intensity variable based on household tobacco expenditure and the average cigarette price for each year of the analysis. This variable accounts for the number of cigarettes consumed by the household during the previous 30 days as a derived (noisy) measure of household smoking intensity that overestimates (underestimates) consumption by those in the highest (lowest) socioeconomic level.

- *Total and specific expenses:* All goods and services, which are classified by the Quality of Life Survey (ECV, the acronym for the Spanish title) into four groups, weekly, monthly, quarterly, and annually, were adjusted to a monthly basis. Weekly expenses consisted of actual or estimated (in cases of free acquisition) spending on food and personal items. Monthly spending covered aggregated, actual, or estimated expenditure on household items such as light bulbs, cleaning items, rent, administration fees, and other items. Quarterly and annual spending involved expenditure on clothing and repairs, improvements to housing, furnishings, and culture and entertainment.

- *Tobacco budget share:* Tobacco expenses were included in the personal spending account, and for the purposes of this study, we constructed a new variable that separated this item from other personal items. Finally, we calculated the share of the budget allocated to expenditure on tobacco using total household expenditure.

- *Annual household income (adjusted for household composition):* Total annual household income is the aggregate of income derived from wages and salaries, capital income (business income, dividend and interest income, and income from other assets), pensions, and expenditure by the government and non-profit institutions serving households that directly benefit households such as health care, childcare, and education. We used the OECD modified equivalent scale to adjust household income. This scale, first proposed by Hagenaars et al. (1994), assigns a value of 1 to the household head, 0.5 to each additional adult member of the household, and 0.3 to each child in the household. Finally, we expressed these figures in log form.

Expenditure categories

- **Food and beverages:** This category includes all bread and cereals, milk and its derivatives, eggs, beef, pork and mutton, goose flesh and chicken meat, fish and other seafood, jam and sausages, potatoes, rice, beans and other grains, ripe and green bananas, vegetables, and fruits, and other forms of nourishment, except expenditure on eating out, which is included in the "Others" category.

- **Tobacco:** Tobacco products, matches, and lighters are included in this category.

- **Alcohol:** This category includes all alcoholic beverages.

- **Clothing:** This category includes veiled stockings for women, clothes, footwear, and all goods/services related to clothing maintenance and repair.

- **Household services:** This category includes a huge variety of services and goods related to the maintenance and functioning of the household, such as house cleaning service per day, electrical goods, administration fees, fuel for cooking, sewerage payments, rent, property tax payments, television, and phone and Internet service payments.

- **Health:** This category includes out-of-pocket expenses for health services, such as affiliation payments or discounts, additional health insurance programs, hospitalization, dentistry, outpatient surgery, medication, and vaccines.

- **Education:** This category includes out-of-pocket expenses for education, such as school fees, uniforms, books and school supplies, transport to and from school, school meals, scholarships, subsides, and student loans.

- **Transport:** This category includes both private and public transport costs, including maintenance, and communication services and goods. This includes the purchase, maintenance, and repair of vehicles for private use, mailing services, fuel and parking costs, bus tickets, and taxi fares.

- **Culture and recreational services:** This category includes newspapers and magazines, entertainment, and books and CDs. It also includes expenditure on trips, such as hotel accommodation and fares.

- **Others:** This category includes a wide variety of goods and services that cannot be classified in any of the previous categories, such as lotteries and other forms of gambling, eating out, toiletries, laundry services outside the home, haircuts and manicures, credit card payments, transfers to other households, home improvements, acquisition of real estate, vehicle tax payments, jewelry, and paintings and other works of art.

Final remarks on data construction and use

To assess variations among similar households, we used propensity score matching, which involved selecting and weighting 2011 non-smokers and 1997 smokers and non-smokers to enable comparison with 2011 smokers. This enabled characteristics such as age, household composition, income, and education level of the household head to remain constant across the four groups.

Appendix B. Robustness

B.1 No matching

Table B.1: SUR Estimates for variation on shares between smokers and non-smokers after Kernel Matching

**Variable**	Alcohol				Others			
	1997	2003	2008	2011	1997	2003	2008	2011
A: Q1 Smokers Share	0.010	0.011	0.006	0.016	0.081	0.069	0.097	0.092
	(0.001)	(0.001)	(0.001)	(0.002)	(0.004)	(0.003)	(0.005)	(0.005)
B: Q5 vs Q1 smokers share: $Q_5^{it} \cdot s_{it}$	0.026	0.015	0.020	0.011	0.052	0.037	0.125	0.152
	(0.005)	(0.003)	(0.004)	(0.004)	(0.017)	(0.010)	(0.016)	(0.014)
C: Q1 Non-smokers Share	0.006	0.005	0.003	0.005	0.083	0.071	0.094	0.095
	(0.001)	(0.001)	(0.001)	(0.001)	(0.003)	(0.001)	(0.002)	(0.002)
D: Q5 vs Q1 non-smokers share: $Q_5^{it} \cdot ns_{it}$	0.017	0.009	0.011	0.010	0.074	0.046	0.135	0.123
	(0.004)	(0.002)	(0.003)	(0.002)	(0.015)	(0.008)	(0.012)	(0.009)
E: Share difference smok. vs non-smok. Q1	0.004	0.006	0.004	0.012	0.012	0.007	-0.003	-0.008
p-val	0.034	0.000	0.045	0.000	0.013	0.020	0.606	0.104
F: Gradient difference smok. vs non-smok. Q1	0.008	0.006	0.010	0.001	-0.021	-0.009	-0.009	0.029
p-val	0.019	0.011	0.012	0.809	0.044	0.166	0.465	0.016

**Variable**	Transport				Housing			
	1997	2003	2008	2011	1997	2003	2008	2011
A: Q1 Smokers Share	0.026	0.046	0.041	0.054	0.120	0.268	0.272	0.203
	(0.002)	(0.002)	(0.004)	(0.003)	(0.007)	(0.005)	(0.009)	(0.008)
B: Q5 vs Q1 smokers share: $Q_5^{it} \cdot s_{it}$	0.017	0.003	0.019	0.017	0.029	0.009	0.040	-0.019
	(0.006)	(0.006)	(0.006)	(0.006)	(0.018)	(0.011)	(0.014)	(0.014)
C: Q1 Non-smokers Share	0.032	0.049	0.049	0.052	0.148	0.314	0.299	0.236
	(0.001)	(0.001)	(0.001)	(0.001)	(0.004)	(0.003)	(0.004)	(0.003)
D: Q5 vs Q1 non-smokers share: $Q_5^{it} \cdot ns_{it}$	0.014	0.002	0.012	0.022	0.022	0.001	0.025	-0.045
	(0.005)	(0.005)	(0.005)	(0.004)	(0.016)	(0.009)	(0.011)	(0.009)
E: Share difference smok. vs non-smok. Q1	-0.008	-0.005	-0.010	0.004	-0.024	-0.029	-0.012	-0.035
p-val	0.001	0.043	0.003	0.298	0.000	0.000	0.130	0.000
F: Gradient difference smok. vs non-smok. Q1	0.004	0.002	0.007	-0.005	0.007	0.009	0.016	0.026
p-val	0.370	0.624	0.167	0.265	0.519	0.199	0.143	0.024

**Variable**	Food				Clothing			
	1997	2003	2008	2011	1997	2003	2008	2011
A: Q1 Smokers Share	0.540	0.437	0.478	0.526	0.105	0.096	0.051	0.057
	(0.008)	(0.005)	(0.011)	(0.010)	(0.009)	(0.005)	(0.005)	(0.004)
B: Q5 vs Q1 smokers share: $Q_5^{it} \cdot s_{it}$	-0.135	-0.075	-0.221	-0.163	0.072	0.020	0.027	0.024
	(0.017)	(0.010)	(0.018)	(0.015)	(0.017)	(0.008)	(0.009)	(0.007)
C: Q1 Non-smokers Share	0.533	0.422	0.494	0.557	0.124	0.099	0.056	0.061
	(0.005)	(0.003)	(0.004)	(0.003)	(0.006)	(0.003)	(0.002)	(0.002)
D: Q5 vs Q1 non-smokers share: $Q_5^{it} \cdot ns_{it}$	-0.126	-0.076	-0.232	-0.168	0.050	0.016	0.024	0.019
	(0.016)	(0.009)	(0.015)	(0.011)	(0.016)	(0.007)	(0.007)	(0.005)
E: Share difference smok. vs non-smok. Q1	0.004	-0.003	-0.021	-0.025	-0.012	-0.005	0.001	-0.002
p-val	0.630	0.587	0.048	0.011	0.237	0.357	0.862	0.602
F: Gradient difference smok. vs non-smok. Q1	-0.009	0.001	0.010	0.005	0.022	0.004	0.004	0.006
p-val	0.398	0.930	0.432	0.690	0.040	0.423	0.473	0.242

**Variable**	Health				Education			
	1997	2003	2008	2011	1997	2003	2008	2011
A: Q1 Smokers Share	0.053	0.028	0.035	0.034	0.039	0.034	0.002	0.000
	(0.004)	(0.002)	(0.004)	(0.003)	(0.003)	(0.002)	(0.001)	(0.001)
B: Q5 vs Q1 smokers share: $Q_5^{it} \cdot s_{it}$	0.014	-0.011	0.013	0.017	-0.002	0.026	0.002	0.005
	(0.009)	(0.005)	(0.008)	(0.007)	(0.008)	(0.005)	(0.002)	(0.002)
C: Q1 Non-smokers Share	0.064	0.030	0.032	0.034	0.040	0.039	0.001	0.002
	(0.003)	(0.001)	(0.002)	(0.001)	(0.002)	(0.001)	(0.000)	(0.000)
D: Q5 vs Q1 non-smokers share: $Q_5^{it} \cdot ns_{it}$	0.005	-0.010	0.021	0.027	-0.016	0.013	0.003	0.008
	(0.008)	(0.004)	(0.007)	(0.005)	(0.006)	(0.005)	(0.001)	(0.002)
E: Share difference smok. vs non-smok. Q1	-0.018	-0.006	-0.001	-0.005	-0.006	-0.006	0.002	-0.000
p-val	0.000	0.004	0.758	0.171	0.140	0.008	0.218	0.802
F: Gradient difference smok. vs non-smok. Q1	0.009	-0.001	-0.008	-0.010	0.013	0.012	-0.001	-0.003
p-val	0.116	0.728	0.185	0.061	0.028	0.002	0.560	0.008

Notes: This table summarises the results with total expenditure net of expenditure on tobacco. Estimates are produced after estimating a SUR directly with ECV 1997, 2003, 2008, and 2011 data. Each set of columns corresponds to a category of spending per year. Quantiles are based on total annual household expenditures adjusted for household composition (Hagennars et al., 1994). In each year, the unconditional shares for smokers and non-smokers from quintile 1 are presented (rows A and C), as well as the difference of these shares for quintile 5 which correspond to equation 1 estimated coefficients conditional on controls (rows B and D). Below them, twotests compare the previous numbers between smokers and non-smokers (A - C, B - D), both of them computed with the estimates of equation 1. Controls include log-expenditures, squared log-expenditures, log-age, female dummy, education level dummies (primary orless [base], secondary, tertiary), a dummy that indicates if the household resides in an urban area, the ratio of the number of childrenunder 5 per adult, household size, and log annual-income adjusted for household composition. Standard errors in parentheses.

B.2 Kernel matching

Table B.2: Matching Sample Balance after kernel matching

		1997		2003		2008		2011	
Variable	Sample	Smoker	Non-Smoker	Smoker	Non-Smoker	Smoker	Non-Smoker	Smoker	Non-Smoker
Age	NM	47.690***	46.467***	46.616***	46.927***	49.271	47.342***	49.939	48.001***
	M	49.806	49.887	49.333	49.321*	49.682	49.467		49.537
Gender (Female=1)	NM	0.205***	0.258	0.272	0.328***	0.228***	0.323***	0.270	0.323***
	M	0.268	0.267	0.249*	0.273	0.254	0.274		0.288*
Primary school	NM	0.842**	0.793	0.634***	0.616***	0.830**	0.752***	0.811	0.700***
	M	0.819	0.816	0.814	0.797*	0.823	0.812		0.799*
Secondary school	NM	0.077	0.100	0.130***	0.154***	0.093	0.147***	0.089	0.153***
	M	0.086	0.088	0.085	0.090	0.088	0.090		0.095
Tertiary school	NM	0.081*	0.107	0.236***	0.230***	0.078***	0.101	0.099	0.147***
	M	0.095	0.096	0.102	0.113**	0.089	0.098		0.106
Zone (Urban=1)	NM	0.540	0.609***	0.773***	0.814***	0.537	0.604***	0.539	0.584***
	M	0.549	0.537	0.552	0.543	0.533	0.553		0.550
Ratio children-under-5/adults	NM	0.801***	0.782***	0.580***	0.637***	0.590***	0.685***	0.515	0.652***
	M	0.534	0.528	0.514	0.531	0.522	0.530		0.547**
Total individuals	NM	4.612***	4.106***	3.922	3.665***	4.226***	3.978	3.919	3.876
	M	3.933	3.910	3.930	3.772***	3.913	3.918		3.841*

Notes: Per variable, the first row corresponds to the sample without matching (NM), and the second to the matched sample (M). Genetic matching with the propensity score, with five neighbours, population size of the optimizer of 10000. Significance of t-test between smokers of each year, and smokers of 2011: * 10%, ** 5%, *** 1%.

Table B.3: SUR Estimates for variation on shares between smokers and non-smokersafter Kernel Matching

Variable	Alcohol				Others			
	1997	2003	2008	2011	1997	2003	2008	2011
A: Q1 Smokers Share	0.012	0.011	0.007	0.016	0.082	0.071	0.096	0.092
	(0.001)	(0.001)	(0.001)	(0.001)	(0.003)	(0.002)	(0.003)	(0.002)
B: Q5 vs Q1 smokers share: $Q_5 \cdot s_{it}$	0.029	0.030	0.022	0.022	0.086	0.084	0.136	0.164
	(0.008)	(0.009)	(0.006)	(0.006)	(0.021)	(0.021)	(0.019)	(0.018)
C: Q1 Non-smokers Share	0.006	0.005	0.003	0.005	0.079	0.070	0.097	0.095
	(0.001)	(0.001)	(0.001)	(0.001)	(0.003)	(0.002)	(0.003)	(0.002)
D: Q5 vs Q1 non-smokers share: $Q_5 \cdot ns_{it}$	0.022	0.014	0.013	0.021	0.115	0.092	0.145	0.138
	(0.007)	(0.006)	(0.005)	(0.006)	(0.019)	(0.017)	(0.017)	(0.014)
E: Share difference smok. vs non-smok. Q1	0.006	0.005	0.004	0.012	0.006	0.005	-0.005	-0.006
p-val	0.038	0.001	0.040	0.000	0.295	0.166	0.371	0.179
F: Gradient difference smok. vs non-smok. Q1	0.007	0.015	0.009	0.001	-0.028	-0.009	-0.009	0.026
p-val	0.232	0.003	0.023	0.853	0.027	0.521	0.478	0.030

Variable	Transport				Housing			
	1997	2003	2008	2011	1997	2003	2008	2011
A: Q1 Smokers Share	0.026	0.043	0.041	0.054	0.161	0.262	0.287	0.203
	(0.002)	(0.002)	(0.002)	(0.002)	(0.006)	(0.003)	(0.005)	(0.004)
B: Q5 vs Q1 smokers share: $Q_5 \cdot s_{it}$	0.002	0.016	0.021	0.021	0.037	-0.014	0.028	-0.021
	(0.008)	(0.011)	(0.007)	(0.007)	(0.021)	(0.017)	(0.018)	(0.015)
C: Q1 Non-smokers Share	0.033	0.047	0.048	0.051	0.184	0.290	0.302	0.236
	(0.002)	(0.002)	(0.002)	(0.002)	(0.006)	(0.003)	(0.005)	(0.004)
D: Q5 vs Q1 non-smokers share: $Q_5 \cdot ns_{it}$	0.004	0.011	0.015	0.026	0.019	0.001	0.019	-0.046
	(0.007)	(0.009)	(0.007)	(0.006)	(0.019)	(0.016)	(0.015)	(0.013)
E: Share difference smok. vs non-smok. Q1	-0.007	-0.005	-0.009	0.004	-0.027	-0.025	-0.007	-0.035
p-val	0.049	0.056	0.005	0.254	0.001	0.000	0.418	0.000
F: Gradient difference smok. vs non-smok. Q1	-0.002	0.005	0.006	-0.006	0.019	-0.016	0.009	0.025
p-val	0.750	0.402	0.205	0.236	0.209	0.191	0.434	0.028

Variable	Food				Clothing			
	1997	2003	2008	2011	1997	2003	2008	2011
A: Q1 Smokers Share	0.509	0.446	0.469	0.526	0.115	0.098	0.053	0.057
	(0.006)	(0.004)	(0.006)	(0.004)	(0.007)	(0.003)	(0.002)	(0.002)
B: Q5 vs Q1 smokers share: $Q_5 \cdot s_{it}$	-0.141	-0.093	-0.240	-0.221	0.053	0.018	0.033	0.033
	(0.022)	(0.018)	(0.022)	(0.018)	(0.026)	(0.014)	(0.009)	(0.007)
C: Q1 Non-smokers Share	0.509	0.448	0.491	0.559	0.115	0.098	0.055	0.061
	(0.006)	(0.004)	(0.006)	(0.004)	(0.007)	(0.003)	(0.002)	(0.002)
D: Q5 vs Q1 non-smokers share: $Q_5 \cdot ns_{it}$	-0.137	-0.108	-0.259	-0.232	0.037	0.016	0.029	0.027
	(0.019)	(0.017)	(0.020)	(0.016)	(0.019)	(0.011)	(0.008)	(0.008)
E: Share difference smok. vs non-smok. Q1	0.004	-0.005	-0.025	-0.030	0.001	-0.002	-0.000	-0.003
p-val	0.710	0.383	0.027	0.002	0.967	0.721	0.920	0.542
F: Gradient difference smok. vs non-smok. Q1	-0.004	0.015	0.019	0.011	0.016	0.002	0.005	0.006
p-val	0.736	0.240	0.170	0.342	0.302	0.802	0.355	0.212

Variable	Health				Education			
	1997	2003	2008	2011	1997	2003	2008	2011
A: Q1 Smokers Share	0.054	0.030	0.031	0.034	0.031	0.029	0.001	0.000
	(0.003)	(0.001)	(0.002)	(0.002)	(0.002)	(0.001)	(0.000)	(0.000)
B: Q5 vs Q1 smokers share: $Q_5 \cdot s_{it}$	0.008	-0.016	0.008	0.015	0.000	-0.002	0.005	0.006
	(0.011)	(0.010)	(0.009)	(0.008)	(0.008)	(0.006)	(0.002)	(0.002)
C: Q1 Non-smokers Share	0.067	0.034	0.033	0.034	0.038	0.033	0.001	0.001
	(0.003)	(0.001)	(0.002)	(0.002)	(0.002)	(0.001)	(0.000)	(0.000)
D: Q5 vs Q1 non-smokers share: $Q_5 \cdot ns_{it}$	-0.003	-0.014	0.017	0.027	-0.015	-0.009	0.005	0.010
	(0.011)	(0.009)	(0.008)	(0.007)	(0.006)	(0.005)	(0.002)	(0.002)
E: Share difference smok. vs non-smok. Q1	-0.015	-0.005	-0.001	-0.003	-0.008	-0.005	0.001	-0.000
p-val	0.003	0.036	0.807	0.445	0.028	0.015	0.494	0.751
F: Gradient difference smok. vs non-smok. Q1	0.010	-0.002	-0.009	-0.012	0.014	0.007	0.000	-0.003
p-val	0.160	0.753	0.117	0.025	0.027	0.073	0.818	0.005

Notes: This table summarises the results with total expenditure net of expenditure on tobacco. Estimates are produced after estimating a SUR using weights produced after a kernel matching using ECV 1997, 2003, 2008, and 2011 data. Each set of columns corresponds to a category of spending per year. Quantiles are based on total annual household expenditures adjusted for household composition (Hagennars et al., 1994). In each year, the unconditional shares for smokers and non-smokers from quintile 1 are presented (rows A and C), as well as the difference of these shares for quintile 5 which correspond to equation 1 estimated coefficients conditional on controls (rows B and D). Below them, two tests compare the previous numbers between smokers and non-smokers (A - C, B - D), both of them computed with the estimates of equation 1. Controls include log-expenditures, squared log-expenditures, log-age, female dummy, education level dummies (primary or less [base], secondary, tertiary), a dummy that indicates if the household resides in an urban area, the ratio of the number of children under 5 per adult, household size, and log annual-income adjusted for household composition. Standard errors in parentheses.
